# Long-Term Clinical Follow-Up of Patients With Chronic Rhinosinusitis

**DOI:** 10.1177/0003489420962822

**Published:** 2020-10-01

**Authors:** Ulrica Thunberg, Amanj Saber, Bo Söderquist, Svante Hugosson

**Affiliations:** 1 Department of Otorhinolaryngology, Örebro University Hospital, Örebro, Sweden; 2 Faculty of Medicine and Health, Örebro University, Örebro, Sweden; 3 Department of Laboratory Medicine, Clinical Microbiology, Örebro University Hospital, Örebro, Sweden

**Keywords:** rhinosinusitis, follow-up, prognosis, quality of life, treatment

## Abstract

**Objective:**

This study comprised a long-term follow-up of a cohort of patients with chronic rhinosinusitis (CRS) regarding clinical features and symptomatology.

**Methods:**

Data from 42 patients with CRS were available from a previous study. Forty of these patients were alive and were contacted for inclusion after approximately 10 years. Patients completed a questionnaire about disease and symptoms, and underwent a clinical examination.

**Results:**

Thirty-four patients (85%) responded and could be included and evaluated. For the participants in this follow-up study median length of time between initial inclusion (C1) and follow-up (C2) was 11 years (range: 8-15). In some patients the CRS shifted phenotype over time, from CRS with nasal polyposis to CRS without nasal polyposis or vice versa. The median total visual analogue score for combined sinonasal symptoms for all patients was statistically significantly reduced at follow-up. For individual patients, scores for nasal congestion, nasal discharge, facial pressure, and hyposmia were also statistically significantly reduced. The most frequently reported symptom-relieving treatments were nasal steroids and saline rinsing of the nose. Self-reported general quality of life was statistically significantly improved at C2 compared to C1.

**Conclusion:**

At long-term follow-up, symptoms were generally reduced and patients reported an improved quality of life. Patients can be given hope for eventual symptom relief. CRS is a chronic condition that seems to harbor the ability to alter its phenotype after several years. Topical corticosteroids and saline rinsing of the nose should be emphasized, since patients consider these treatments to be of high value.

## Introduction

Chronic rhinosinusitis (CRS) is probably one of the most common healthcare problems among adults in the western world. Patients with CRS are regular visitors to ENT clinics and require medication, sometimes surgery, and always encouragement. There is a great need for better understanding of this disease. CRS is an overall term for a heterogeneous condition characterized by inflammation and remodeling of the mucosal tissue of the nose and paranasal sinuses. Different groups of CRS can be distinguished on the basis of phenotype, including CRS with nasal polyposis (CRSwNP) and CRS without nasal polyposis (CRSsNP). The disease shows a spectrum of symptoms such as nasal congestion, nasal discharge, headache, cough, fatigue, facial fullness, bad breath, and changes in smell and taste. A combination of symptoms lasting longer than 3 months is the basis for a CRS diagnosis.^1,2^ Symptoms of CRS often last for years, and studies have confirmed that the disease is associated with a significant reduction in quality of life (QOL).^3-5^ Patients’ QOL and symptomatology are in parity with other chronic disease, such as cardiac disease.^6^

The main choice for surgical intervention is functional endoscopic sinus surgery to accomplish disease control in severe cases and when conservative treatment is insufficient. The crucial point in the treatment is conservative therapy, aimed at local disease control and reduction of sinonasal symptoms. Topical application of corticosteroids is used together with nasal saline irrigation and sometimes nasal antihistamines. Sometimes oral corticosteroids are used. There is no evidence-based guidance for antibiotic treatment, but a general strategy is to administer short-course antibiotics according to microbiological cultures and mainly for acute exacerbation. The present study comprises an approximately 11-year follow-up of a cohort of CRS patients regarding clinical features, treatment, and self-assessment of symptomatology in a long-term perspective.

## Materials and Methods

### Patients and Collection of Clinical Data

The study population was based on 42 CRS patients from a previous study.^7^ These patients were re-evaluated after approximately 11 years, at which point 40 of them were still alive and could be invited to participate. The first data collection is referred to as time point C1 and the follow-up as C2. The follow-up study was yet not planned at C1. Two physicians specialized in otorhinolaryngology (UT and SH) performed all inclusion procedures. All patients invited to participate had CRS for more than 1 year (range: 1-9 years, median: 3 years) prior to inclusion at C1, and were included in conjunction with sinus surgery or when visiting the ENT outpatient clinic for regular control. The CRS diagnosis was based on history, clinical examination, and computed tomography scan according to the definitions and guidelines of the American Academy of Otolaryngology—Head and Neck Surgery.^2^ These inclusion criteria match the definition from the position paper on rhinosinusitis guidelines^1^ prepared by the European Academy of Allergology and Clinical Immunology (EAACI) and approved by the European Rhinologic Society (ERS). The patients from C1 were contacted and followed-up during 2017 to 2019. All participants were legally of full age (>18 years) at C1, and provided written informed consent to participate in the study. Information about age, gender, acetylsalicylic acid (ASA) intolerance, asthma, and nasal polyps were collected from the patients by questionnaire or from medical records when necessary. Endoscopic visualization of the nasal cavity was performed. The presence or absence of nasal polyps was documented, and polyp grade was classified as 0 to 3: 0 = no polyps, 1 = polyp(s) visible in the middle meatus, 2 = polyp(s) protruding from the middle meatus into the nasal cavity, 3 = large polypoid masses partially or totally occluding the nasal cavity.^8^

## Symptoms and Quality of Life

The overall degree of symptoms was evaluated via a 6-parameter symptom score questionnaire from the Swedish Rhinologic Society, with answers given on a stepless visual analogue scale (VAS) symbolizing the range between “absence of symptoms” and “worst symptoms possible.” This procedure was used in order to avoid clustering of scores around a preferred numeric value. Each patient filled in the VAS at both time points (C1 and C2). A ruler was used to convert the respondents’ markings to the corresponding numbers between 0 (no symptoms) and 10 (the worst symptoms possible). Symptoms evaluated were nasal congestion, nasal discharge, loss of smell, facial pain or pressure, coughing, and QOL in terms of fatigue. A total score was calculated for each individual, with a maximal (worst) value of 60 points. A median score was also calculated, with a value of ≤3 regarded as mild, >3 to 7 as moderate, and ≥7 as severe, in concordance with previous studies.^1,9^ To strengthen comparison of answers, the same form for collection of VAS was used at both C1 and C2. Patients were invited to report a maximum of 3 symptoms that they regarded as worst from a list of eleven: fever, bad breath, toothache, fatigue, headache, and bad smell in the nose, loss of smell, cough, facial pressure, secretion, and nasal congestion. They were also asked which symptom-reliving treatment(s) for sinus problems had helped them the most over the past 10 years; no examples of treatment were provided, and patients were free to give more than 1 treatment option. QOL in relation to sinus disease was evaluated by asking patients to assess their current QOL in comparison to their QOL 10 years previously, with 3 answer alternatives: “no change,” “better,” or “worse.”

## Statistical Analysis

The participants’ demographic characteristics and symptom scores are presented using descriptive statistical methods. Ordinal data are summarized as medians with corresponding interquartile ranges (IQR) where suitable, and categorical data are presented as percentages. Differences between groups were tested using the Mann-Whitney *U*-test. Wilcoxon’s test was used for paired data and 2 related groups. Bivariate logistic regression was applied to data, using CRSwNP and CRSsNP as the dependent variable and symptom score as independent variable. A 2-sided *P-*value <.05 was considered statistically significant. All statistical analyses were conducted using version 22 of IBM SPSS Statistics.

## Results

### Patient Characteristics

**Figure 1.**
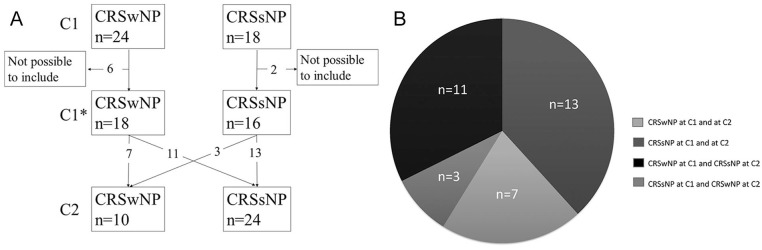
Distribution of chronic rhinosinusitis with nasal polyposis (CRSwNP) and chronic rhinosinusitis without nasal polyposis (CRSsNP) at C1 and C2. *Note*. Time points C1 (initial inclusion), time point C2 (follow-up). *Number of patients included from C1.

Two of the 42 CRS patients included at C1 were deceased at C2. Of the remaining 40 patients, 34 (85%; 22 women and 12 men) responded at C2 and were included for this follow-up study. Eight patients were not possible to include. The 6 patients who did not respond all had CRSwNP while both the deceased patients had CRSsNP. The median length of time between C1 and C2 was 11 years (range: 8-15). Mean age was 52 years at C1 and 63 years at C2. Nasal polyposis was present in 18/34 patients at C1 (10 grade 1 and 8 grade 2) and 10 at C2 (8 grade 1 and 2 grade 2). No patients had grade 3 polyps at either time point. The distribution of CRSwNP and CRSsNP at C1 and C2 is presented in Figure 1. There was no AFRS or CF in our study group. Five of the 8 patients who reported ASA intolerance at C1 had CRSwNP. Two patients reported the development of ASA intolerance over time; both had persisting CRSsNP and neither of them reported asthma. One patient with nasal polyposis at C1 was found without nasal polyposis at C2 and reported recovery from ASA intolerance. Twelve patients reported asthma at C1; all except 1 had nasal polyposis (92%). At C2, 3 of these 12 patients reported asthma and 2 of the 3 had recovered from nasal polyposis. Samter’s triad (CRSwNP, ASA intolerance, and asthma) was present in 4/34 patients at C1; none of them had Samter’s triad at C2, though, 1 still had asthma and all 4 still had ASA intolerance. Of the eleven patients (32%) who said they were smokers or exposed to passive smoking at C1, 6 of them reported smoking cessation at C2. One patient had started to smoke, and so 6 patients reported smoking or exposure to smoke at C2. There was no statistical difference in total symptom score between smokers or individuals with passive smoking exposure compared to non-smokers (*P* = .8). Patients clinical characteristics presented in Table 1.

**Table 1.** Clinical Data for All Patients with Chronic Rhinosinusitis (CRS) with Nasal Polyposis (CRSwNP) and CRS Without Nasal Polyposis (CRSsNP) Observed at 2 Time Points (C1 Initial Inclusion, C2 Follow-Up a Decade Later).

All CRS	C1	C2	*P* value
	n = 34	n = 34	
Age, mean	52 (34-77)	63 (45-88)	NA
Sex (female), n	22	22	1.0
ASA intolerance, n	8	9	1.0
Asthma, n	12	3	.01
Exposed to smoking daily, n	11	6	.1
Nasal congestion^a^	5.9	3.7	.001
Nasal discharge^a^	7.3	5.4	.03
Hyposmia^a^	6.7	5.1	.001
Facial pressure^a^	5.6	4.1	.005
Cough^a^	4.3	2.9	.07
QOL in terms of fatigue^a^	6.9	4.7	.005
CRSwNP	C1 n = 18^b^	C2 n = 10	*P* value
Sex (female), n	10	4	.5
ASA intolerance, n	5	2	1.0
Asthma, n	10	1	.07
Nasal congestion^a^	5.7	2.8	.02
Nasal discharge^a^	7.3	4.8	.1
Hyposmia^a^	7.2	4.6	.1
Facial pressure^a^	5.2	3.0	.07
Cough^a^	4.3	1.5	.02
QOL in terms of fatigue^a^	6.7	4.5	.1
CRSsNP	C1 n = 16	C2 n = 24	*P* value
Sex (female), n	12	18	1.0
ASA intolerance, n	3	7	.7
Asthma, n	2	2	.8
Nasal congestion^a^	6.1	4.1	.02
Nasal discharge^a^	7.5	5.6	.05
Hyposmia^a^	6.2	5.3	.6
Facial pressure^a^	6.2	4.6	.2
Cough^a^	4.3	3.4	.5
QOL in terms of fatigue^a^	7.1	4.8	.001

*Note*. The *P* value was determined by Fisher exact test or Mann-Whitney test.

Abbreviation: NA: not applicable.

a Mean score on VAS (max 10 points).

b Two symptom score missing.

### Self-Reported Symptomatology

**Figure 2.**
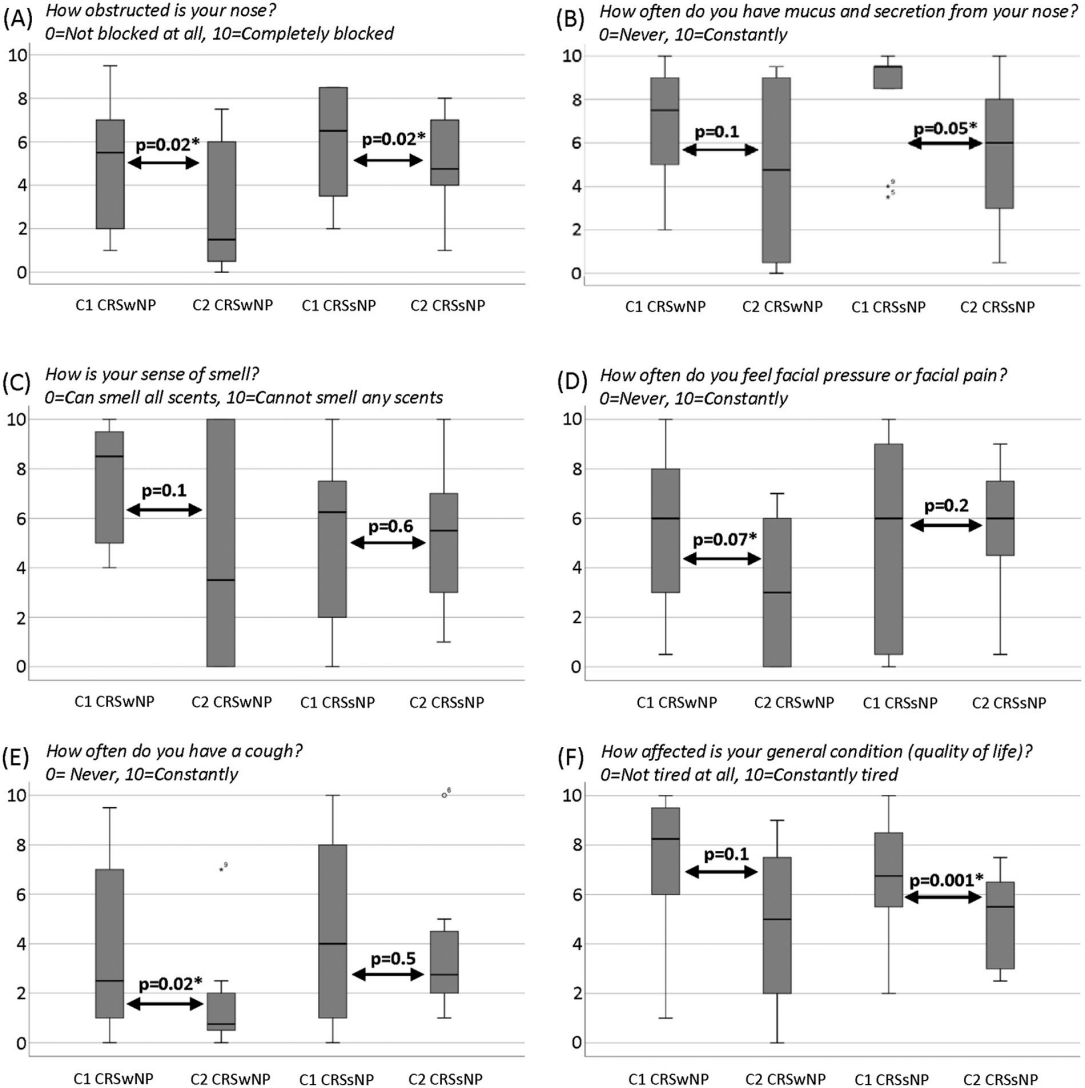
Patients’ VAS assessments of symptoms (range: 0-10) at C1and C2. Data for questions A-F are split by group: chronic rhinosinusitis with nasal polyposis (CRSwNP) and chronic rhinosinusitis without nasal polyposis (CRSsNP). *Note*. Time points C1 (initial inclusion), time point C2 (follow-up) 2 patients with CRSsNP did not give any answers. For comparison Mann-Whitney *U*-test was used. The horizontal lines represent median values of reported VAS, the bottom and top lines of the boxes represent the 25th and 75th percentiles, the whiskers extending below and above the boxes represent the minimum and maximum values, and the circles above some of the boxes represent outlier cases.

**Figure 3.**
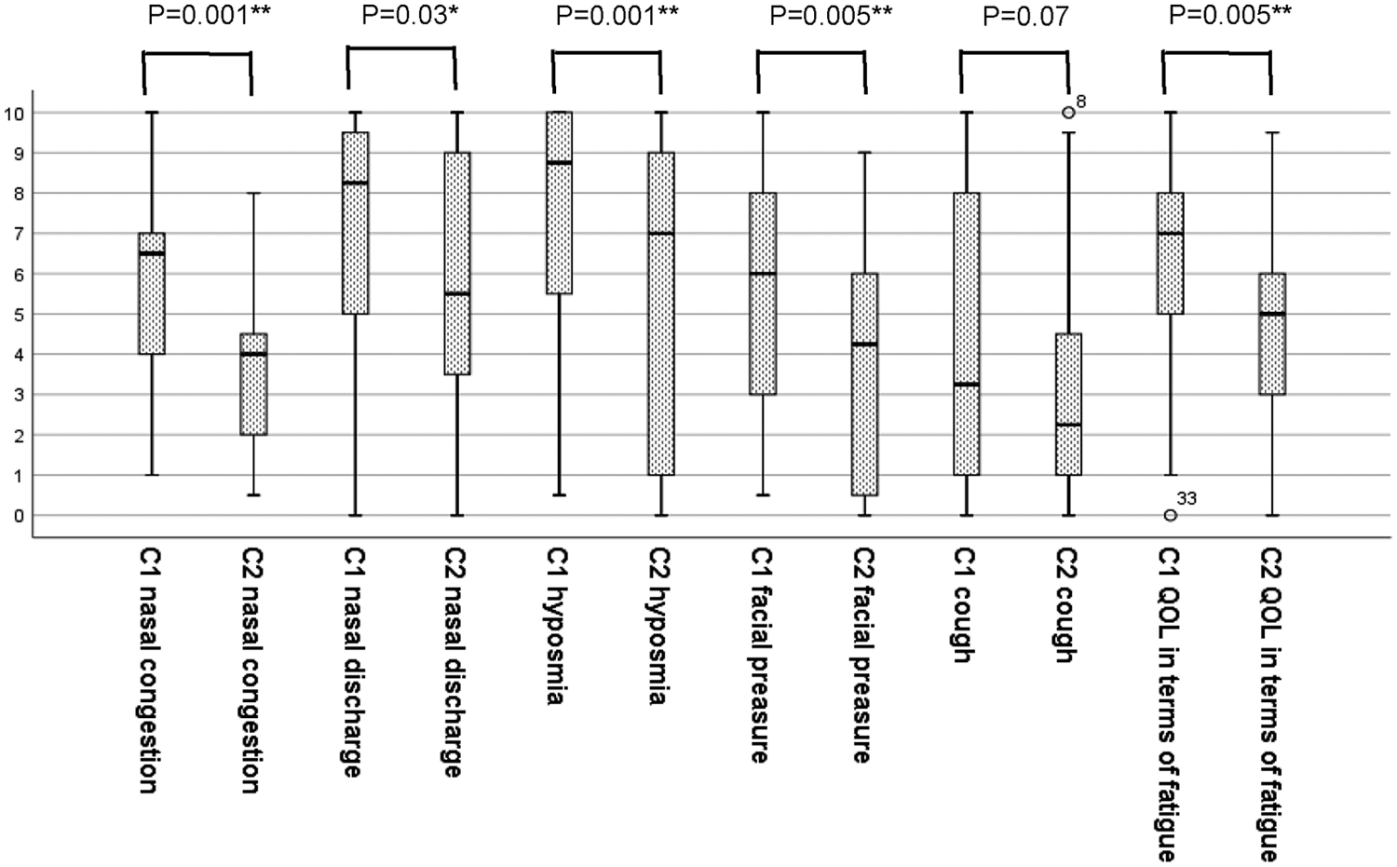
Differences in the individual patients’ assessments of symptoms according to VAS (range: 0-10) for questions A-F described in Figure 2 at C1 and C2. *Note*. Time points C1 (initial inclusion), time point C2 (follow-up) The horizontal lines represent median values of reported VAS, the bottom and top lines of the boxes represent the 25th and 75th percentiles, the whiskers extending below and above the boxes represent the minimum and maximum values, and the circles above 2 of the boxes represent outlier cases. The paired-samples Wilcoxon test was used. *P*-values are for change in symptom score between time points C1 and C2 in individuals. Two of the 34 patients did not give an answer.

VAS scores for severity of symptoms in the CRSwNP and CRSsNP groups at C1 and C2 are shown in Figure 2. The median total VAS score (max 60) for combined sinonasal symptoms for all patients was 38 (range: 9.5-54) at C1 and 26 (range: 1.5-44.5) at C2. The reduction was statistically significant (*P* = .001). Comparing the groups, QOL in terms of fatigue showed statistically significant improvement after 10 years among CRSsNP patients (*P* = .001) but not among their CRSwNP counterparts (*P* = .1). There was no significant difference in change in total score between men and women. According to the VAS classification, 3% of the patients had mild, 23% moderate, and 68% severe disease at C1. Two patients did not fill in the VAS. At C2, 15% of the patients had mild, 62% moderate, and 24% severe disease. Table 2 shows the worst symptoms at C1 and C2 according to the patient estimation. As noted earlier, each of the 34 patients was permitted to choose up to 3 symptoms, giving a maximum possible number of 102 choices. At C1, there were 94 choices while at C2 there were 79; in addition, more patients refrained from choosing at C2 than at C1. Scores for nasal congestion, facial pressure, and hyposmia showed a statistically significant reduction for individuals (Figure 3). There was no significant difference in answers about loss of smell among the patient group who moved from CRSwNP to CRSsNP. Individual patients’ QOL in terms of fatigue was statistically significantly better at C2 than at C1 (*P* = .005). Regarding QOL now as compared to 10 years ago in terms of sinus disease, 18 of the 34 patients (28 % CRSwNP, 72% CRSsNP) thought it was better, 6 (50% CRSwNP, 50% CRSsNP) thought it was worse, and 10 (20% CRSwNP, 80% CRSsNP) considered it unchanged. In the logistic regression none of the symptoms showed statistical significance as predictor for CRSwNP or CRSsNP.

**Table 2.** Individual Chronic Rhinosinusitis Patients’ Choices of Up to 3 Symptoms that they Considered to be Worst, Chosen from a Specified List of 11 Symptoms.

Symptom	Number of times chosen (max 3 choices per patient) C1 = 94 chosen symptoms C2 = 79 chosen symptoms	
	C1	C2
Nasal congestion	24	17
Nasal discharge	22	10
Loss of smell	19	12
Facial pressure	17	13
Fatigue	8	4
Headache	2	7
Cough	2	7
Bad smell in nose	0	4
Toothache	0	2
Bad breath	0	2
Fever	0	1
No symptoms reported	8	20

*Note*. Number of patients choosing a specific symptom at time points C1 (initial inclusion) and C2 (follow-up).

### Treatment

Patients’ answers regarding symptom-reducing treatment are shown in Table 3. The most frequently reported symptom-relieving treatments were nasal steroids and saline rinsing of the nose, particularly among patients with CRSsNP. Three patients reported no need for treatment at all. Eighteen patients (53%) had no sinus surgery before C1, and 17 (50%) had no sinus surgeries between C1 and C2. Three patients had sinus surgery more than 3 times over the past 15 years; 2 of them had ASA intolerance and asthma at C1, and 1 of these reported recovery from asthma at C2.

**Table 3.** Individual Patients’ Answers at Time Point C2 (Follow-Up) to the Open Question Asking What Symptom-Relieving Treatment (s) for Sinus Problems had Helped Them the Most Over the Past 10 Years.

Symptom-relieving treatment	Number of times chosen		
	CRSwNP n = 10	CRSsNP n = 24	All CRS n = 34
Saline rinsing	2	12	14
Nasal corticosteroids	3	8	11
Alpha-receptor agonists	1	3	4
Oral corticosteroids	2	1	3
Sinus surgery	1	2	3
Phenylpropanolamine	0	2	2
Self-care with laser	0	1	1
Physical exercise	0	1	1
Antibiotics	0	2	2
Nasal oil	0	1	1
Antihistamine	0	0	0
No need of treatment	2	1	3

*Note*. Patients were free to write more than 1 treatment option. No patients reported more than 3 specific treatments; a majority reported 2 treatments. Split data for chronic rhinosinusitis with nasal polyposis (CRSwNP) and chronic rhinosinusitis without nasal polyposis (CRSsNP). Three patients said they had no need of treatment.

## Discussion

This study comprised a long-term follow-up of a cohort of patients with CRS. Few other studies exist with such a long follow-up of CRS patients, and so the present study adds valuable information in terms of clinical features, symptoms, and therapeutic effect in a long-term perspective. QOL in terms of fatigue showed statistically significant improvement after 10 years among CRSsNP patients (*P* = .001) but not among their CRSwNP counterparts (*P* = .1). When patients were asked to choose their 3 worst symptoms from a list of eleven, the most frequently chosen were nasal discharge, nasal congestion, hyposmia, and facial pressure. All of these symptoms were significantly reduced between C1 and C2 for the individual patients. Calus et al showed that smell disturbance and nasal congestion were the predominant symptoms preoperatively, and that these improved significantly after 12 years.^10^ In the present study, 23% of patients did not choose any worst symptoms at C1, and this proportion rose to 61% at C2. It is not clear whether the symptoms actually diminished or whether the patients simply got used to them. Nevertheless, what is important is how the patients experience their symptoms. Eighteen of the 34 patients (53%) considered that their QOL related to their sinus disease was better than 10 years ago. Our study collected data by means of self-application of the instrument, in order to remove any potential bias caused by an interviewer. In addition, at C2 patients were not given any information about their answers from C1.

Since the initial collection at C1, different instruments for measuring health-related QOL in evaluating the outcome of CRS have gained attention. Sahlstrand-Johnson et al^11^ showed in a study of 207 patients referred for endoscopic sinus surgery that the higher the VAS score, the worse the patient scored in the questionnaires used for estimation of QOL (SNOT-22, SF-36, and HADS). The authors suggested that as a consequence, a simple question such as “How troublesome are your rhinosinusitis symptoms?” could be used to estimate severity of disease and provide a basis for decisions about sinus surgery. This finding supports using the VAS as a tool to estimate severity of disease.^11^ In our study the same questionnaire was used at both time points, which strengthens the abovementioned findings. Based on VAS score, patients were divided into having mild, moderate, and severe disease according to a previously described method.^1,9^ Severe disease was present in 68% at C1 and only 24% at C2. Patients, especially those in the CRSsNP group, considered saline irrigation and nasal corticosteroids to be the most valuable treatments for symptom relief. The use of nasal corticosteroids has been shown effective as a CRS treatment,^12-14^ and combined surgery and corticosteroid treatment in nasal polyposis has been shown to reduce symptoms in long-term follow-up.^15^ Patients in the present study did not regard antibiotics as an important help in reducing symptoms. A recent systematic review examining the effects of cigarette smoke on CRS patients in the United States strongly suggested that both active and passive smoke exposure increase the risk of CRS.^16^

Of the 34 included patients, 18 had CRSwNP at C1, and 11 (61%) of these had recovered from polyposis at C2. Interestingly, 3 (19%) of the 16 patients with CRSsNP at C1 had developed nasal polyps at C2. The high recovery rate from nasal polyposis is probably due to surgical intervention and corticosteroid treatment. A 12-year follow-up of CRSwNP after endoscopic sinus surgery found that nasal polyps were absent in 40% of patients.^10^ Frequency of nasal polyposis varies substantially in different studies, due to factors such as variation in postoperative treatment, surgical technique, and duration of follow-up.

We found ASA intolerance (self-reported or stated in the medical record) in 5/18 (28%) patients with CRSwNP at C1. Another questionnaire study reported a 40% incidence of ASA intolerance in patients with CRSwNP with comorbid asthma.^17^ In our material, 1 patient developed polyps over the study period but was assessed as having recovered from ASA intolerance. Information about ASA intolerance, smoking, and asthma was collected from patients’ medical records and the questionnaire. No objective tests were performed; these tests could have strengthened our results. Fewer patients reported asthma at C2 than at C1, which might reflect improvement in their CRS. It has been shown that more extended polyps in CRS are associated with more severe asthma in CRS patients with comorbid asthma.^18^ A systematic review of 24 articles searching for evidence linking allergy to CRSsNP and CRSwNP, concluded that the role of allergy in both phenotypes is still controversial and that the level of evidence is low.^19^

Classification of CRS into subgroups such as CRSsNP and CRSwNP based on phenotype alone is now regarded as insufficient, and the identification of different inflammation profiles for endotypes is gaining interest as a future model for classification.^20-23^ Cluster analysis based on biomarkers such as cytokine expression, chemokines, cellular infiltration, and tissue remodeling pattern has been suggested.^22^ Studies of inflammatory cell profiles indicate that patients with CRSwNP and CRSsNP are immunologically distinguishable; a majority of CRSsNP patients show a Th1-biased inflammatory response, while CRSwNP patients primarily show a Th2-biased response. A recent European study analyzing 14 biomarkers were analyzed in sinonasal tissue identified 10 clusters showed IL-5 levels to be important.^22^ Nasal polyposis tissue has an increased immune response to *S. aureus* enterotoxins, resulting in more pronounced eosinophilic inflammation and higher total IgE production in the tissue of patients affected by CRSwNP.^24,25^ For example, SAE-specific IgE-positive nasal polyps show more severe eosinophilic inflammation compared to controls. Severity in this context is based on a greater synthesis of IL-5 and eotaxin. These patients more often have asthma and/or ASA intolerance.^26^ Significantly higher levels of IgE directed against SAE have been found in sera of CRS patients compared with sera of healthy controls.^27^ The aim of this study was to evaluate symptomatology and clinical features in patients with CRS, but no information about endotypes was available. It would have been of interest to know the inflammation profile in the present study, given that phenotype changes were seen after many years in some cases.

Due to the limited number of patients in our study, we cannot rule out the possibility that some selection bias occurred at initial inclusion. All the 6 non-responders had CRSwNP which might affect the results. Since the initial inclusion a decade ago did not include studies of endotypes we do not know whether the switch of phenotypes after many years was due to a change of biomarkers and endotype which can be regarded as a limitation. Additional information about grading of disease severity for period at C1 could have been valuable. Some questionnaire data were missing for 2 patients with CRSsNP, which could have been different from those collected and analyzed. Nevertheless, such a long-term follow-up is rare, and 85% of patients were re-evaluated at follow-up.

## Conclusion

Symptoms were generally reduced and VAS QOL in terms of fatigue for individual patients was improved after approximately 11 years (range: 8-15). When considering only their CRS, 28% of patients with CRSwNP and 72% of patients with CRSsNP regarded their QOL as better than it was 10 years ago, indicating that patients with CRSsNP might have a greater chance of symptom relief than their CRSwNP counterparts in a long-term perspective. CRS seems to harbor the ability to alter its phenotype after several years, but further long-term studies are needed on phenotypes and endotypes. Topical corticosteroids and saline rinsing of the nose should be emphasized, since the patients considered these treatments to be of high value in the long term.

## References

[1] Fokkens WJ Lund VJ Mullol J, et al. European Position Paper on Rhinosinusitis and Nasal Polyps 2012. Rhinol Suppl. 2012;23:3 p preceding table of contents, 1-298. 22764607

[2] Benninger MS Ferguson BJ Hadley JA, et al. Adult chronic rhinosinusitis: definitions, diagnosis, epidemiology, and pathophysiology. Otolaryngol Head Neck Surg. 2003; 129(suppl 3):S1-S32. 12958561 10.1016/s0194-5998(03)01397-4

[3] Erskine S Hopkins C Kumar N, et al. A cross sectional analysis of a case-control study about quality of life in CRS in the UK; a comparison between CRS subtypes. Rhinology. 2016;54(4):311-315. 27315942 10.4193/Rhin15.361

[4] Sahlstrand-Johnson P Hopkins C Ohlsson B Ahlner-Elmqvist M. The effect of endoscopic sinus surgery on quality of life and absenteeism in patients with chronic rhinosinuitis - a multi-centre study. Rhinology. 2017;55(3):251-261. 28624844 10.4193/Rhin16.126

[5] Hoehle LP Phillips KM Bergmark RW Caradonna DS Gray ST Sedaghat AR. Symptoms of chronic rhinosinusitis differentially impact general health-related quality of life. Rhinology. 2016;54(4):316-322. 27665614 10.4193/Rhin16.211

[6] Gliklich RE Metson R. The health impact of chronic sinusitis in patients seeking otolaryngologic care. Otolaryngol Head Neck Surg. 1995;113(1):104-109. 7603703 10.1016/S0194-59989570152-4

[7] Thunberg U Hugosson S Monecke S Ehricht R Soderquist B. Molecular characteristics of Staphylococcus aureus associated with chronic rhinosinusitis. APMIS. 2015;123(1):37-44. 25131615 10.1111/apm.12299

[8] Danielsen A Olofsson J. Endoscopic endonasal sinus surgery: a review of 18 years of practice and long-term follow-up. Eur Arch Otorhinolaryngol. 2006;263(12):1087-1098. 16937113 10.1007/s00405-006-0129-4

[9] Lim M Lew-Gor S Darby Y Brookes N Scadding G Lund VJ. The relationship between subjective assessment instruments in chronic rhinosinusitis. Rhinology. 2007;45(2):144-147. 17708462

[10] Calus L Van Bruaene N Bosteels C, et al. Twelve-year follow-up study after endoscopic sinus surgery in patients with chronic rhinosinusitis with nasal polyposis. Clin Transl Allergy. 2019;9:30. 31249662 10.1186/s13601-019-0269-4 PMC6570859

[11] Sahlstrand-Johnson P Ohlsson B Von Buchwald C Jannert M Ahlner-Elmqvist M. A multi-centre study on quality of life and absenteeism in patients with CRS referred for endoscopic surgery. Rhinology. 2011;49(4):420-428. 21991567 10.4193/Rhino11.101

[12] Harvey R Hannan SA Badia L Scadding G. Nasal saline irrigations for the symptoms of chronic rhinosinusitis. Cochrane Database Syst Rev. 2007(3):CD006394. 10.1002/14651858.CD006394.pub2 17636843

[13] Harvey RJ Snidvongs K Kalish LH Oakley GM Sacks R. Corticosteroid nasal irrigations are more effective than simple sprays in a randomized double-blinded placebo-controlled trial for chronic rhinosinusitis after sinus surgery. Int Forum Allergy Rhinol. 2018;8(4):461-470. 29394004 10.1002/alr.22093

[14] Pundir V Pundir J Lancaster G, et al. Role of corticosteroids in Functional Endoscopic Sinus Surgery–a systematic review and meta-analysis. Rhinology. 2016;54(1):3-19. 26970247 10.4193/Rhin15.079

[15] Bonfils P. Evaluation of the combined medical and surgical treatment in nasal polyposis. I: functional results. Acta Otolaryngol. 2007;127(4):436-446. 17453467 10.1080/00016480600895078

[16] Christensen DN Franks ZG McCrary HC Saleh AA Chang EH. A systematic review of the association between cigarette smoke exposure and chronic rhinosinusitis. Otolaryngol Head Neck Surg. 2018;158(5):801-816. 29460678 10.1177/0194599818757697

[17] Jarvis D Newson R Lotvall J, et al. Asthma in adults and its association with chronic rhinosinusitis: the GA^2^LEN survey in Europe. Allergy. 2012;67(1):91-98. 22050239 10.1111/j.1398-9995.2011.02709.x

[18] Micheletto C Visconti M Trevisan F Tognella S Bertacco S Dal Negro RW. The prevalence of nasal polyps and the corresponding urinary LTE4 levels in severe compared to mild and moderate asthma. Eur Ann Allergy Clin Immunol. 2010;42(3):120-124. 20648775

[19] Wilson KF McMains KC Orlandi RR. The association between allergy and chronic rhinosinusitis with and without nasal polyps: an evidence-based review with recommendations. Int Forum Allergy Rhinol. 2014;4(2):93-103. 24395734 10.1002/alr.21258

[20] Bachert C Akdis CA. Phenotypes and emerging endotypes of chronic rhinosinusitis. J Allergy Clin Immunol Pract. 2016; 4(4):621-628. 27393777 10.1016/j.jaip.2016.05.004

[21] Avdeeva K Fokkens W. Precision medicine in chronic rhinosinusitis with nasal polyps. Curr Allergy Asthma Rep. 2018; 18(4):25. 29574586 10.1007/s11882-018-0776-8 PMC5866836

[22] Tomassen P Vandeplas G Van Zele T, et al. Inflammatory endotypes of chronic rhinosinusitis based on cluster analysis of biomarkers. J Allergy Clin Immunol. 2016;137(5):1449-1456.e4. 10.1016/j.jaci.2015.12.1324 26949058

[23] Akdis CA Bachert C Cingi C, et al. Endotypes and phenotypes of chronic rhinosinusitis: a PRACTALL document of the European Academy of Allergy and Clinical Immunology and the American Academy of Allergy, Asthma & Immunology. J Allergy Clin Immunol. 2013;131(6):1479-1490. 23587334 10.1016/j.jaci.2013.02.036 PMC4161279

[24] Van Crombruggen K Zhang N Gevaert P Tomassen P Bachert C. Pathogenesis of chronic rhinosinusitis: inflammation. J Allergy Clin Immunol. 2011;128(4):728-732. 21868076 10.1016/j.jaci.2011.07.049

[25] Shiobara N Suzuki Y Aoki H, et al. Bacterial superantigens and T cell receptor beta-chain-bearing T cells in the immunopathogenesis of ulcerative colitis. Clin Exp Immunol. 2007; 150(1):13-21. 17614973 10.1111/j.1365-2249.2007.03443.x PMC2219284

[26] Bachert C Gevaert P Holtappels G Johansson SG van Cauwenberge P. Total and specific IgE in nasal polyps is related to local eosinophilic inflammation. J Allergy Clin Immunol. 2001;107(4):607-614. 11295647 10.1067/mai.2001.112374

[27] Tieu DD Kern RC Schleimer RP. Alterations in epithelial barrier function and host defense responses in chronic rhinosinusitis. J Allergy Clin Immunol. 2009;124(1):37-42. 19560577 10.1016/j.jaci.2009.04.045 PMC2802265

